# Rational, computer-aided design of multi-target ligands

**DOI:** 10.1186/1758-2946-3-S1-P10

**Published:** 2011-04-19

**Authors:** J Achenbach, E Proschak

**Affiliations:** 1 Pharmaceutical Chemistry, Goethe University, 60438 Frankfurt am Main, Germany

## 

Over the past two decades the “one drug – one target – one disease” concept became the prevalent paradigm in drug discovery. The main idea of this approach is the identification of a single protein target whose inhibition leads to a successful treatment of the examined disease. The predominant assumption is that highly selective ligands would avoid unwanted side effects caused by binding to secondary non-therapeutic targets.

In recent years the results of post-genomic and network biology showed that proteins rarely act in isolated systems but rather as a part of a highly connected network [1]. In addition this connectivity leads to more robust systems that cannot be interfered by the inhibition of a single target of that network and consequently might not lead to the desired therapeutic effect [2]. Furthermore studies prove that robust systems are rather affected by weak inhibitions of several parts than by a complete inhibition of a single selected element of that system [3].

Therefore there is an increasing interest in developing drugs that take effect on multiple targets simultaneously but is concurrently a great challenge for medicinal chemists. There has to be a sufficient activity on each target as well as an adequate pharmacokinetic profile [4]. Early design strategies tried to link the pharmacophors of known inhibitors, however these methods often lead to high molecular weight and low ligand efficacy.

We present a new rational approach based on a retrosynthetic combinatorial analysis procedure [5] on approved ligands of multiple targets. These RECAP fragments are used to design a large combinatorial library containing molecules featuring chemical properties of each ligand class. The molecules are further validated by machine learning models, like random forests and self-organizing maps, regarding their activity on the targets of interest.
